# Validating a breast cancer score in Spanish women. The MCC-Spain study

**DOI:** 10.1038/s41598-018-20832-0

**Published:** 2018-02-14

**Authors:** Trinidad Dierssen-Sotos, Inés Gómez-Acebo, Camilo Palazuelos, Pablo Fernández-Navarro, Jone M Altzibar, Carmen González-Donquiles, Eva Ardanaz, Mariona Bustamante, Jessica Alonso-Molero, Carmen Vidal, Juan Bayo-Calero, Adonina Tardón, Dolores Salas, Rafael Marcos-Gragera, Víctor Moreno, Paz Rodriguez-Cundin, Gemma Castaño-Vinyals, María Ederra, Laura Vilorio-Marqués, Pilar Amiano, Beatriz Pérez-Gómez, Nuria Aragonés, Manolis Kogevinas, Marina Pollán, Javier Llorca

**Affiliations:** 1 CIBER Epidemiología y Salud Pública (CIBERESP), Madrid, Spain; 2 University of Cantabria – IDIVAL, Santander, Spain; 3 Cancer and Environmental Epidemiology Unit, National Center for Epidemiology, Carlos III Institute of Health, Madrid, Spain; 4 Cancer Epidemiology Research Group, Oncology and Hematology Area, IIS Puerta de Hierro (IDIPHIM), Madrid, Spain; 5 Breast Cancer Screening Programme, Basque Health Department, Osakidetza, Spain; 6 Grupo de Investigación Interacciones Gen-Ambiente y Salud, Universidad de León, León, Spain; 7 Navarra Public Health Institute, Navarra, Spain; 8 IdiSNA, Navarra Institute for Health Research, Pamplona, Spain; 9 ISGlobal Centre for Research in Environmental Epidemiology (CREAL), Barcelona, Spain; 10 Centre for Genomic Regulation (CRG), the Barcelona Institute of Science and Technology, Barcelona, Spain; 11 Universitat Pompeu Fabra (UPF), Barcelona, Spain; 12 Cancer Prevention and Control Program, Catalan Institute of Oncology-IDIBELL, L’Hospitalet de Llobregat, Barcelona, Spain; 13 Complejo Hospitalario Universitario de Huelva, Centro de Investigación en Salud y Medio Ambiente (CYSMA), Universidad de Huelva, Huelva, Spain; 14 IUOPA, Universidad de Oviedo, Asturias, Spain; 15 Valencia Cancer and Public Health Area, FISABIO – Public Health, Valencia, Spain; 16 General Directorate Public Health, Valencian Community, Valencia, Spain; 17 Epidemiology Unit and Girona Cancer Registry, Oncology Coordination Plan, Department of Health, Autonomous Government of Catalonia and Descriptive Epidemiology, Genetics and Cancer Prevention Group [Girona Biomedical Research Institute (IdIBGi)], Catalan Institute of Oncology, Girona, Spain; 18 Department of Clinical Sciences, Faculty of Medicine, University of Barcelona, Barcelona, Spain; 19 University Hospital Marques de Valdecilla – IDIVAL, Santander, Spain; 20 IMIM (Hospital Del Mar Medical Research Institute), Barcelona, Spain; 21 Public Health Division of Gipuzkoa, BioDonostia Research Institute, San Sebastian, Spain; 22 School of Public Health, Athens, Greece

**Keywords:** Cancer epidemiology, Breast cancer

## Abstract

A breast-risk score, published in 2016, was developed in white-American women using 92 genetic variants (GRS92), modifiable and non-modifiable risk factors. With the aim of validating the score in the Spanish population, 1,732 breast cancer cases and 1,910 controls were studied. The GRS92, modifiable and non-modifiable risk factor scores were estimated via logistic regression. SNPs without available genotyping were simulated as in the aforementioned 2016 study. The full model score was obtained by combining GRS92, modifiable and non-modifiable risk factor scores. Score performances were tested via the area under the ROC curve (AUROC), net reclassification index (NRI) and integrated discrimination improvement (IDI). Compared with non-modifiable and modifiable factor scores, GRS92 had higher discrimination power (AUROC: 0.6195, 0.5885 and 0.5214, respectively). Adding the non-modifiable factor score to GRS92 improved patient classification by 23.6% (NRI = 0.236), while the modifiable factor score only improved it by 7.2%. The full model AUROC reached 0.6244. A simulation study showed the ability of the full model for identifying women at high risk for breast cancer. In conclusion, a model combining genetic and risk factors can be used for stratifying women by their breast cancer risk, which can be applied to individualizing genetic counseling and screening recommendations.

## Introduction

Breast cancer is the most frequent type of cancer in women worldwide and a main cause of cancer death in developed countries^1^. Epidemiological research has led to the identification of several risk factors (age at menarche, parity, age at first full-term pregnancy, age at menopause), most of them associated with estrogen production^2,3^. A number of risk factors are related with lifestyle (tobacco smoking, alcohol consumption, overweight or obesity), although their importance seems to be smaller than the estrogen/reproductive life-associated risk factors^4^. Known risk factors can only explain about 40% of breast cancer risk.

A few highly penetrant genetic variants, like those in BRCA1 and BRCA2 genes, have been proved to increase breast cancer risk^5,6^; although their low prevalence hardly allows them to explain around 5% cases of breast cancer^7^, carrying any of these variants puts women in such a high risk that screening practices have been adapted in carrier women and even oophorectomy or early mastectomy can be considered in some cases in the absence of breast cancer diagnosis^8^. With the advent of next generation sequencing techniques, an increasing number of low-penetrant genetic variants are being identified as related to breast cancer^7^ and some polygenic tests have been marketed intending to identify women at high risk of breast cancer, although their clinical relevance is uncertain.

In this way, Maas *et al*. presented a breast cancer risk model among white women in the United States, which included modifiable and non-modifiable risk factors, as well as a genetic risk score with information from 92 genetic variants^9^. The goal of this article is to validate Maas *et al*.’s model in a Spanish case-control study.

## Methods

### Study design and population

The Multi Case-Control (MCC-Spain) study is a population-based case-control study of common tumors in Spain and has been described elsewhere. It has been carried out in 23 hospitals and primary care centers in 12 Spanish provinces and assesses five types of cancer (colorectal, breast, stomach, prostate and chronic lymphocytic leukemia) using the same series of population controls for all cases^10^. Cases and controls were recruited between September 1st, 2008 and December 31st, 2013.

We included 1,732 incident cases of breast cancer in women and 1,910 controls in ten participating centers (Asturias, Barcelona, Cantabria, Girona, Gipuzkoa, Huelva, León, Madrid, Navarra and Valencia). Cases were aged 20–85 and with a new pathology-confirmed diagnosis of breast cancer living in the catchment area of each hospital at least 6 months prior to the diagnosis. Controls were women without history of breast cancer living in the same catchment area as cases; they were randomly selected from the rosters of General Practitioners at the Primary Health Centers. Controls were frequency-matched to cases by 5-year age groups and study area. Response rates were 72% among cases and 52% among controls.

The study was carried out according to Spanish laws on biomedical research. All procedures were performed with the ethical standards of the institutional and/or national research committee and with the 1964 Helsinki declaration and its later amendments.

The Ethics Committees of the participating hospitals (Ethical Committee of Clinical Research of Barcelona, Cantabria, Girona, Gipuzkoa, Huelva, León, Principado de Asturias, Madrid, Navarra and Valencia) approved the study protocols, and participants provided written informed consent at the time of enrollment.

### Data collection

Participants were interviewed face-to-face by trained interviewers with a comprehensive epidemiological questionnaire that assessed socio-demographic information, personal and family history of cancer, anthropometric data, alcohol consumption, smoking habits, reproductive and medical history, and family history. Participant’s weight was recorded by self-report, as estimated one year before diagnosis for cases and for controls. Body mass index (BMI) was estimated from self-reported weight and height one year before the diagnosis for cases and one year prior to the interview for controls. Blood samples were obtained following the study protocol. Elapsed time between breast cancer diagnosis and interview was 117 days on average.

### Genotyping

The genotyping was performed for 1,138 cases and 1,239 controls using the Infinium Human Exome BeadChip (Illumina, San Diego, USA) that includes >200,000 coding markers plus 5,000 additional custom SNPs selected from previous GWAS or in genes of interest.

### Genetic score

In order to construct the genetic score, we consider two SNP sets. The first set included 24 SNPs identified from Breast and Prostate Cancer Cohort Consortium (BPC3 study) (Supplementary Table 1)^11–13^. 17 of them were included in the performed genotyping; the remaining 7 (rs10069690, rs10941679, rs17530068, rs1250003, rs10483813, rs6504950 and rs2284378) were imputed using SNPSTATs^14,15^. The 24-SNP genetic score (GRS24) was estimated by the addition of the beta coefficients as obtained in BPC3 study^13^. Barrdahl *et al*.^13^ considered rs10483813 as surrogate for rs999737 as they are in strong linkage disequilibrium; therefore, we excluded rs10483813 from GRS24 in order to do not double the weight of this locus. As genotyping was not available for 594 cases and 671 controls, we simulated GRS24 in them using the method suggested by Chatterjee *et al*.^16^ as explained in the following paragraph.

The second set of SNPs included 68 identified in Breast Cancer Association Consortium (BCAC) and Collaborative Oncological Gene-Environment Study (COGS) (Supplementary Table 1). They were not included in the genotyping, so the 68-SNP genetic score (GRS68) was simulated with the Chatterjee *et al*. method^16^, as performed by Maas *et al*.^9^ when developing the score we are trying to validate. In brief, GRS68 was obtained using the reported beta coefficients (=log odds ratios) and conditional on case-control status and family history of breast cancer as:

1 $$P(GRS{68}_{i}|D=d,\,Family\,H=h) \sim N(\mu ,{\sigma }_{GRS-68}^{2})$$

where $=d\times {\sigma }_{GRS68}^{2}+\frac{1}{2}\times h\times {\sigma }_{GRS68}^{2}$, ${\sigma }_{GRS68}^{2}=\sum _{k}2{\hat{\beta }}_{k}^{2}{f}_{k}(1-{f}_{k})$, subindex *i* refers to the participants included in the study and *k* refers to the SNPs included in the model, ${\hat{\beta }}_{k}$ are the estimates of the log odds ratios, and *f*_*k*_ are the risk allele frequencies. ${\hat{\beta }}_{k}$ and *f*_*k*_ were obtained from the BCAC^17,18^.

Finally, a 92-SNP genetic score (GRS92) was obtained by adding GRS24 plus GRS68. Supplementary Table 2 displays R^2^ as a measure of linkage disequilibrium between the SNPs located in the same chromosome, according to the European population in the 1000 Genome Project and were obtained from https://analysistools.nci.nih.gov/LDlink/?tab=home.

#### Modifiable risk factor score

The modifiable risk factor score (MRFS) included BMI, menopausal hormone therapy, level of alcohol consumption, and smoking status. Alcohol use and BMI were categorized as in Maas *et al*.^9^ (Supplementary Table 3). In order to build MRFS, we carried out a multivariate logistic regression analysis including the above indicated modifiable risk factors. Then, MRFS was obtained by adding the estimated beta coefficients of the above indicated risk factors, every beta being adjusted for each other factor.

#### Non-modifiable risk factor score

The non-modifiable risk factor score (NMRFS) included family history, age at first birth, parity, age at menarche, height, menopausal status, and age at menopause. According to Maas *et al*.^9^, age at first birth and parity were considered “non-modifiable” as it is unlikely that women modify these factors because of their breast cancer risk. Age at menarche, age at first birth, height and age at menopause were categorized as in Maas *et al*.^9^ (Supplementary Table 3). NMRFS was built in the same way that MRFS.

#### Full model

The full model (FM) was obtained as the addition of GRS92 + MRFS + NMRFS.

### Statistical analysis

The independence among the scores was tested using the Pearson correlation coefficient. Differences in scoring between cases and controls were tested with the Student-t test. In order to analyze the association among scores and breast cancer, scores were categorized into deciles; then, a logistic regression model was constructed for each score, with decile 1 as reference. ROC curves and their area under the curve (AUROC) were obtained for each logistic model.

For analyzing whether a score adds or not to the predictive ability of another score, we estimated the net reclassification improvement (NRI), the integrated discrimination improvement (IMI) and the improvement in the AUROC. The goal for a score is to classify breast cancer cases and controls adequately; when two scores -say GRS24 and GRS92- are compared, GRS92 would classify some cases better than GRS24 and some others worse; likewise, GRS92 would classify some controls better and some others worse. NRI^19^ is the net sum of classifying cases and controls better using GRS92 rather than GRS24:

2 $$\begin{array}{rcl}NRI & = & P(cases\,classified\,better\,with\,GRS{92})\\  &  & -P(cases\,classified\,worse\,with\,GRS{92})\\  &  & +P(controls\,classified\,better\,with\,GRS{92})\\  &  & -P(controls\,classified\,worse\,with\,GRS{92})\end{array}$$

It is noteworthy that NRI can only be applied when both scores are nested one in each other, as it is the case between GRS24 and GRS92, because GRS92 equals GRS24 plus GRS68. In this article, we use the continuous version of NRI^20^.

While NRI measures the improvement in classification, IMI estimates the improvement in predicted probability. A better score is expected to predict higher probabilities in cases and lower probabilities in controls than a worse score. Therefore, IMI is defined as^19^:

3 $$\begin{array}{rcl}IMI & = & P(Breast\,cancer\,estimated\,with\,GRS{92}\,in\,cases)\\  &  & -P(Breast\,cancer\,estimated\,with\,GRS{24}\,in\,cases)\\  &  & +P(Breast\,cancer\,estimated\,with\,GRS{24}\,in\,controls)\\  &  & -P(Breast\,cancer\,estimated\,with\,GRS{92}\,in\,controls)\end{array}$$

Finally, in order to explore the ability of the scores to identify women at high risk of breast cancer, we simulated the expected breast cancer incidence rate according to their scoring in GRS92, NMRFS, MRFS and full model. For instance, the simulation for GRS92 was built as follows: First, a logistic regression model was estimated using GRS92 as regressor and breast cancer as event, obtaining a *β* coefficient (=log (OR)). Second, incidence rates by 5-year age groups were obtained from Globocan^1,21^; these rates were attributed to patients with the average GRS92. Lastly, incidence rates for each patients were simulated by multiplying the average age-specific incidence rate times exp[β*(GRS92 – average GRS92)]. Similar simulations were performed for NMRFS, MRFS and full model. All statistical analyses were performed with the software Stata 14/SE (Stata Co., College Station, TX, US).

## Results

**Figure 1:**
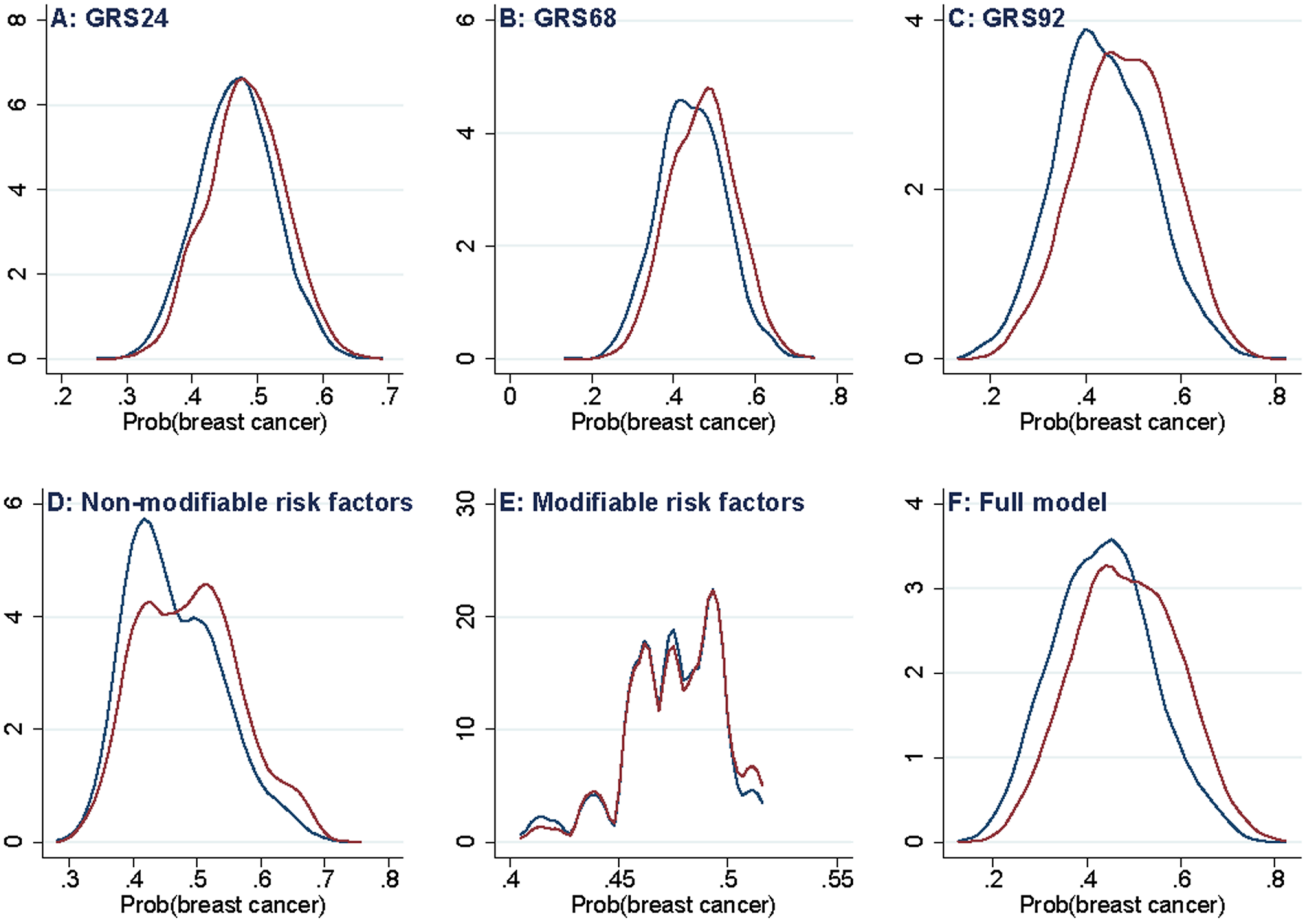
Kernel density plots on distribution of genetic and risk factor scores in breast cancer cases (red line) and controls (blue line).

1,732 breast cancer cases and 1,910 controls were included in the analysis; their main characteristics are reported in Supplementary Table 4. Compared to controls, cases were 2.6 younger in average, they have about twice the probability of having a first degree relative affected of breast cancer, their age at menarche was a bit earlier and their age at menopause was later, and they have less children. There were no differences in height, BMI, alcohol consumption and age at first delivery. The distributions of genetic risk scores with 24 [GRS24], 68 [GRS68] and 92 [GRS92] SNPs; modifiable risk factor score [MRFS]; nonmodifiable risk factor score [NMRFS], and full model [FM] are reported in Fig. 1. All scores show a high degree of overlapping between cases and controls, being MRFS the less discriminant score. BC cases scored higher than controls in each risk score (Supplementary Table 5). GRS92, MRFS and NMRFS were not linearly dependent from each other as their Pearson correlation coefficient was <0.06 (Supplementary Table 6).

The association between GRS92 and breast cancer is displayed in Table 1, where the odds ratio increased decile by decile; patients in the 10^th^ decile had 3.8 the odds of breast cancer than patients in the first decile and double the odds than patients around the median of GRS92 distribution (i.e.: patients in 5^th^ or 6^th^ deciles). The area under the ROC curve (AUROC) was 0.6195; GRS24 and GRS68 -the two components of GRS92- had smaller prediction ability, with AUROC being equal to 0.5676 and 0.5936, respectively (Supplementary Figure 1).

**Table 1:** Relationship among breast cancer and different scores. Odds ratios per decile of each score.

Decile	GRS92	NMRFS	MRFS	Full model
1	1 (ref.)	1 (ref.)	1 (ref.)	1 (ref.)
2	1.27 (0.90–1.78)	0.90 (0.67–1.22)	1.03 (0.79–1.34)	1.82 (1.29–2.56)
3	1.22 (0.87–1.71)	1.18 (0.88–1.58)	1.05 (0.76–1.46)	1.54 (1.09–2.17)
4	1.79 (1.28–2.49)	1.02 (0.75–1.37)	1.16 (0.88–1.53)	2.56 (1.83–3.60)
5	2.00 (1.43–2.78)	1.36 (1.01–1.83)	0.83 (0.64–1.08)	2.12 (1.51–2.98)
6	2.17 (1.56–3.03)	1.63 (1.21–2.20)	1.33 (0.96–1.84)	2.08 (1.48–2.92)
7	2.35 (1.69–3.27)	1.59 (1.18–2.14)	1.11 (0.85–1.45)	2.34 (1.67–3.29)
8	2.54 (1.82–3.54)	2.00 (1.48–2.70)	1.06 (0.78–1.44)	3.28 (2.34–4.60)
9	3.82 (2.74–5.35)	2.03 (1.50–2.73)	1.14 (0.87–1.49)	4.45 (3.16–6.26)
10	3.80 (2.72–5.32)	2.43 (1.79–3.29)	1.46 (1.08–1.97)	5.70 (4.02–8.07)

GRS92: Genetic Risk Score with 92 SNPs.

NMRFS: Non-modifiable Risk Factor Score.

MRFS: Modifiable Risk Factor Score.

Nonmodifiable risk factors show a dose-response relationship with breast cancer from the fifth decile on; the first four deciles, however, do not exhibit any increase in risk (Table 1). The AUROC was 0.5885 (Supplementary Figure 1).

Modifiable risk factor score had little association with breast cancer (Table 1); only patients in the 10^th^ decile had an odds ratio around 1.5 respect to patients in the first decile. Its ROC curve was hardly over the diagonal, displaying small discrimination power (AUROC = 0.5214) (Supplementary Figure 1).

The improvement in risk prediction when adding more component scores from GRS24 on was measured with three indicators: net reclassification index (NRI) (equation (2)), integrated discrimination improvement (IDI) (equation (3)) and improvement in AUROC, whose results are displayed in Table 2. Adding GRS68 to GRS24 (i.e.: constructing GRS92) improved patient classification by 28.2% (NRI = 0.282) and the difference in the probabilities of suffering breast cancer between cases and controls improved by 2.7% (IDI = 0.027). Adding NMRFS up to GRS92 still improved patient classification by 23.6%, in spite of moderate improvements in IDI (0.015) and AUROC (0.0141). Adding MRFS to GRS92, however, only scored 7.2% in NRI with marginal enhancements in IDI and AUROC. Being low the betterment of MRFS over GRS92, most of it seems to be redundant with NMRFS as adding MRFS to NMRFS hardly improved patient classification (NRI = 0.017), discrimination (IDI = 0.000) and AUROC (−0.0013).

**Table 2:** Improvement in risk prediction when adding more component scores.

Base score	Enhanced score	Net Reclassification Improvement	Integrated Discrimination Improvement	Improvement in AUROC	p value*
**GRS24**	**GRS24 + GRS68 = GRS92**	0.282	0.027	0.0484	<0.001
**GRS92**	**GRS92 + NMRFS**	0.236	0.015	0.0141	0.01
**GRS92**	**GRS92 + MRFS**	0.072	0.003	0.0024	0.37
**NMRFS**	**NMRFS + MRFS = RFS**	0.017	0.000	−0.0013	0.18

^*^p value for the improvement in AUROC.

GRS24: Genetic Risk Score with 24 SNPs.

GRS68: Genetic Risk Score with 68 SNPs.

GRS92: Genetic Risk Score with 92 SNPs.

NMRFS: Non-modifiable Risk Factor Score.

MRFS: Modifiable Risk Factor Score.

RFS: Risk Factor Score.

The full model, thus, mainly reflects the combination of the genetic score GRS92 and the non-modifiable risk score (NMRFS). Its relationship with breast cancer stepped up by each decile (Table 1); the AUROC reached 0.6244 (Supplementary Figure 1).

**Figure 2:**
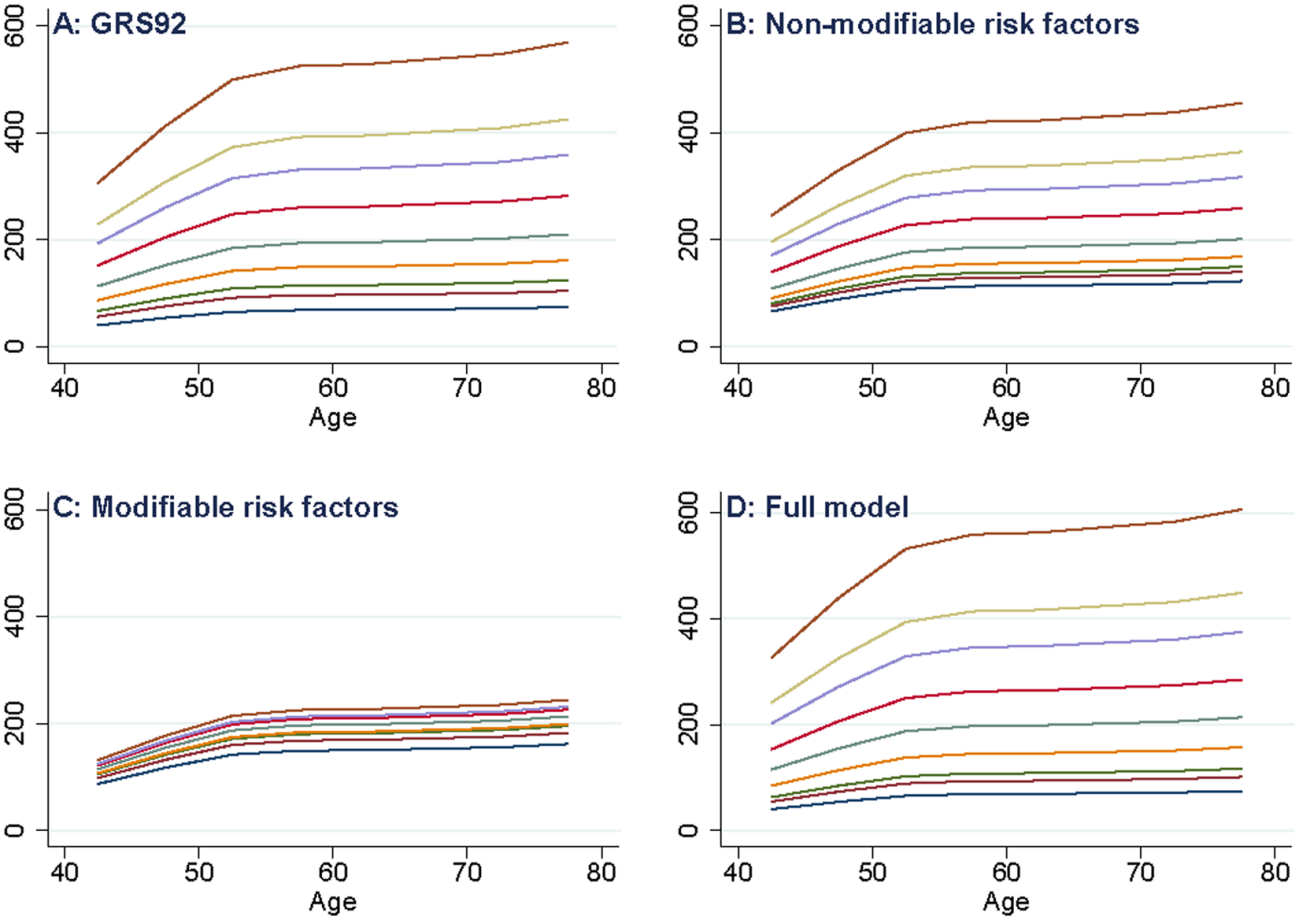
Projected breast-cancer incidence rate by age, according to the percentiles of the scores. (**A**) Genetic score; (**B**) Non-modifiable risk factors; (**C**) Modifiable risk factors; (**D**) Full model. In each graphic, lines from bottom to top represent percentiles 1, 5, 10, 25, 50, 75, 90, 95 and 99.

Figure 2 exhibits the expected breast cancer incidence rates by age, according to GRS92, NMRFS, MRFS and full model. Lines represent percentiles 1, 5, 10, 25, 50, 75, 90, 95 and 99 of each score distribution. Figure 2A–D are represented in the same Y scale in order to easily identify which models are more able to classify patients according to their risk: the more separated the lines are, the higher the model ability to classify patients. GRS92 (Fig. 2A), NMRFS (Fig. 2B) and especially full model (Fig. 2D) allowed for identifying patients in high risk of breast cancer.

## Discussion

In this case-control study, we have found that a model for breast cancer risk stratification developed in white women in the United States^9^ similarly performs in the Spanish population.

### Relative relevance of GRS, NMRFS, MRFS

According to our results, modifiable risk factors do not discriminate well between breast cases and controls, which questions our ability to implement breast cancer primary prevention policies. Of note, several modifiable factors (namely, alcohol consumption, BMI and tobacco smoking) have been identified as risk factors for many other frequent diseases (e.g.: cardiovascular diseases, lung cancer and other cancers); thus, general recommendations for moderating alcohol consumption, avoiding tobacco smoking and maintaining BMI in its optimal range are considered among the most important health promotion recommendations, despite we do not observe that these factors can discriminate between breast cases and controls.

### Consequences for screening

The US Preventive Services Task Force recommends biennial breast cancer screening with mammography for all women aged 50 to 74 “who are not at high risk of breast cancer because of a known underlying genetic mutation”^22^. GRS92, NMRFS and full model, however, could be useful in stratifying women according to their breast cancer risk. Women scoring in the 10^th^ decile in GRS92 or full model could have double the risk of women with the average scoring and four- to six-fold the risk of women in the first decile, which makes it sensible to use these scores for individualizing the breast cancer screening recommendations. Genetic risk is already being the basis for changing screening periodicity; for instance, women carrying mutations in the gene BRCA1 have about 4-fold the risk of the average women, which supports performing screen mammography every six months^23^ and could even sustain recommendations for oophorectomy and/or mastectomy on an individual basis. In a similar way, a two-fold increase in risk over the average could lead to shorten the usual two-year interval for mammography in women scoring high in GRS92 or in the full model; alternatively, women scoring high in GRS92 or in the full model could benefit from initiating screening mammography before being 50; in this way, results from a simulation study^24^ suggest that women with two-fold to four-fold increased risk for breast cancer could be annually screened starting at age 40 with similar harm-to-benefit ratio than the biennial screening starting at age 50 for the average-risk women. Likewise, women in the first decile have about half the risk of the average women, which could induce to reconsider the balance of harms and benefits they have when being screened and, thus, to discuss the possibility of widening their mammography interval, delaying their starting age or even reconsidering the utility the screening program has for them.

### Consequences for communicating risk

Genetic counseling has been available since testing for BRCA1 and BRCA2 was developed about 20 years ago^25^. New sequencing technologies make polygenic panel tests accessible^26,27^; applying them to risk assessment would allow genetic counseling to go further than a few highly-penetrant variants, which opens new ways for advising women before breast cancer can develop. Moreover, genetic scores on breast cancer and non-modifiable risk factors seem to be independent from each other; according to our results, stratifying breast cancer risk combining both genetic score (GRS92) and non-modifiable risk factors (NMRFS) improves patient classification by 28%. From a woman’s point of view, it is of no relevance whether her [possible] breast cancer would be caused by a sum of genetic variant or non-modifiable risk factors or a combination of both; in this way, it could be time for moving from genetic counseling towards risk counseling, irrespective of the factors related to that risk. Maas *et al*.’s model^9^, which we have here validated in a Spanish population, could be a well-founded instrument for doing it.

### Limitations

Our study has some limitations. First, genotyping was available only for 65% of our patients; therefore, GRS24 had to be imputed in the remaining 35%. In order to validate this imputation, we carried out a sensitivity analysis on the GRS24 – breast cancer relationship in participants with and without genotyping data (Supplementary Table 7); OR by deciles were very similar in both groups, reinforcing the way genotype was imputed. Genotyping of the SNPs included in GRS68, however, was not available at all, so GRS68 was completely imputed as it actually was in the paper were the score was developed^9^. Second, when constructing GRS92, rs10483813 was excluded because of its almost perfect correlation with rs999737; apart from it, only two other SNPs (rs6678914 and rs4245739) displayed R^2^ with each other greater than 0.3 (Supplementary Table 2), involving some degree of linkage disequilibrium; however, we consider that their correlation was not high enough to exclude one of them in spite of admitting some redundancy degree. Third, a main concern in our results was the weak breast cancer - modifiable risk factors association; of note, reproductive and lifestyle factors have been measured by self-reporting; in this way, data are exposed to recall bias. As breast cancer participants were aware of their condition, we cannot rule out that their reports could be more biased that those of controls; this could be especially considered when reporting unhealthy lifestyles such as alcohol consumption or tobacco smoking, eventually leading to downward OR estimations. On the other hand, hormone replacement therapy has been used for less than 10% Spanish women^28^, which makes it difficult to find a relevant impact on breast cancer risk. Fourth, we limited our analysis to validate Maas *et al*.’s model in our case-control study; therefore, we did not go further than their article^13^ in exploring interactions between genetic and non-genetic risk factors, which -on the other hand- could be limited given the sample size our study has. Fifth, other risk factor models for BC have been published; for instance, Rosner and Colditz developed^29^ and refined^30^ a cumulative risk model based on the Nurses’ Health Study. Rosner – Coldtiz model, however, does not include genetic variants, which is a main point in Maas *et al*.’s score; therefore, we have not conducted a specific comparison between them.

In conclusion, when validating a breast cancer model, we have found that adding a non-modifiable risk factor score to a polygenic score can largely improve patient’s risk stratification. Its potential utilities would include risk-based screening programs or changes in genetic/risk counseling on breast cancer.

## Electronic supplementary material

Supplementary material
