# Author Correction: Validating a breast cancer score in Spanish women. The MCC-Spain study

**DOI:** 10.1038/s41598-020-62511-z

**Published:** 2020-04-01

**Authors:** Trinidad Dierssen-Sotos, Inés Gómez-Acebo, Camilo Palazuelos, Pablo Fernández-Navarro, Jone M. Altzibar, Carmen González-Donquiles, Eva Ardanaz, Mariona Bustamante, Jessica Alonso-Molero, Carmen Vidal, Juan Bayo-Calero, Adonina Tardón, Dolores Salas, Rafael Marcos-Gragera, Víctor Moreno, Paz Rodriguez-Cundin, Gemma Castaño-Vinyals, María Ederra, Laura Vilorio-Marqués, Pilar Amiano, Beatriz Pérez-Gómez, Nuria Aragonés, Manolis Kogevinas, Marina Pollán, Javier Llorca

**Affiliations:** 1 CIBER Epidemiología y Salud Pública (CIBERESP), Madrid, Spain; 2 University of Cantabria – IDIVAL, Santander, Spain; 3 Cancer and Environmental Epidemiology Unit, National Center for Epidemiology, Carlos III Institute of Health, Madrid, Spain; 4 Cancer Epidemiology Research Group, Oncology and Hematology Area, IIS Puerta de Hierro (IDIPHIM), Madrid, Spain; 5 Breast Cancer Screening Programme, Basque Health Department, Osakidetza, Spain; 6 Grupo de Investigación Interacciones Gen-Ambiente y Salud, Universidad de León, León, Spain; 7 Navarra Public Health Institute, Navarra, Spain; 8 IdiSNA, Navarra Institute for Health Research, Pamplona, Spain; 9 ISGlobal Centre for Research in Environmental Epidemiology (CREAL), Barcelona, Spain; 10 Centre for Genomic Regulation (CRG), the Barcelona Institute of Science and Technology, Barcelona, Spain; 11 Universitat Pompeu Fabra (UPF), Barcelona, Spain; 12 Cancer Prevention and Control Program, Catalan Institute of Oncology-IDIBELL, L’Hospitalet de Llobregat, Barcelona, Spain; 13 Complejo Hospitalario Universitario de Huelva, Centro de Investigación en Salud y Medio Ambiente (CYSMA), Universidad de Huelva, Huelva, Spain; 14 IUOPA, Universidad de Oviedo, Asturias, Spain; 15 Valencia Cancer and Public Health Area, FISABIO – Public Health, Valencia, Spain; 16 General Directorate Public Health, Valencian Community, Valencia, Spain; 17 Epidemiology Unit and Girona Cancer Registry, Oncology Coordination Plan, Department of Health, Autonomous Government of Catalonia and Descriptive Epidemiology, Genetics and Cancer Prevention Group [Girona Biomedical Research Institute (IdIBGi)], Catalan Institute of Oncology, Girona, Spain; 18 Department of Clinical Sciences, Faculty of Medicine, University of Barcelona, Barcelona, Spain; 19 University Hospital Marques de Valdecilla – IDIVAL, Santander, Spain; 20 IMIM (Hospital Del Mar Medical Research Institute), Barcelona, Spain; 21 Public Health Division of Gipuzkoa, BioDonostia Research Institute, San Sebastian, Spain; 22 School of Public Health, Athens, Greece

Correction to: *Scientific Reports* 10.1038/s41598-018-20832-0, published online 14 February 2018

The original version of this Article contained a typographical error in the spelling of the author Gemma Castaño-Vinyals, which was incorrectly given as Gemma Castaño Vinyals.

In addition, the Supplementary Information file also contained a typographical error in the spelling of the author Jessica Alonso-Molero, which was incorrectly given as Jessica Alonso.

These errors have now been corrected in the PDF and HTML versions of the Article, and in the accompanying Supplementary Information file.

