# Supplementary material for: Validating a breast cancer score in Spanish women. The MCC-Spain study
Source: Sci Rep. 2018 Feb 14;8:3036. doi: 10.1038/s41598-018-20832-0 (PMC5813036; doi:10.1038/s41598-018-20832-0)

TITLE PAGE

Validating a breast cancer score in Spanish women. The MCC-Spain study.

Authors: Trinidad Dierssen-Sotos[1,2], Inés Gómez-Acebo[1,2], Camilo Palazuelos[2], Pablo Fernández-Navarro[1,3,4], Jone M Altzibar[1,5], Carmen González-Donquiles[1,6], Eva Ardanaz[1,7,8], Mariona Bustamante[1,9,10,11], Jessica Alonso-Molero[2], Carmen Vidal[12], Juan Bayo-Calero[13], Adonina Tardón[1,14], Dolores Salas[15,16], Rafael Marcos-Gragera[17], Víctor Moreno[1,12,18], Paz Rodriguez-Cundin[19], Gemma Castaño-Vinyals[1,9,11,20], María Ederra[1,7,8], Laura Vilorio-Marqués[6], Pilar Amiano[1,21], Beatriz Pérez-Gómez[1,3,4], Nuria Aragonés[1,3,4], Manolis Kogevinas[1,9,11,20,22], Marina Pollán[1,3,4], Javier Llorca[1,2]

Affiliations:

1: CIBER Epidemiología y Salud Pública (CIBERESP), Spain.

2: University of Cantabria – IDIVAL, Santander, Spain.

3: Cancer and Environmental Epidemiology Unit, National Center for Epidemiology, Carlos III Institute of Health. Madrid, Spain.

4: Cancer Epidemiology Research Group, Oncology and Hematology Area, IIS Puerta de Hierro (IDIPHIM), Madrid, Spain

5: Breast Cancer Screening Programme, Osakidetza, Basque Health Department, Spain.

6: Grupo de Investigación Interacciones Gen-Ambiente y Salud. Universidad de León, León, Spain.

7: Navarra Public Health Institute, Navarra, Spain.

8: IdiSNA, Navarra Institute for Health Research, Pamplona, Spain.

9: ISGlobal Centre for Research in Environmental Epidemiology (CREAL), Barcelona, Spain

10. Centre for Genomic Regulation (CRG), the Barcelona Institute of Science and Technology, Barcelona, Spain.

11.Universitat Pompeu Fabra (UPF), Barcelona, Spain.

12: Cancer Prevention and Control Program, Catalan Institute of Oncology-IDIBELL, L'Hospitalet de Llobregat, Spain.

13: Complejo Hospitalario Universitario de Huelva, Centro de Investigación en Salud y Medio Ambiente (CYSMA), Universidad de Huelva, Huelva, Spain.

14: IUOPA, Universidad de Oviedo, Asturias, Spain.

15: Valencia Cancer and Public Health Area, FISABIO – Public Health, Valencia, Spain.

16: General Directorate Public Health, Valencian Community. Spain.

17: Epidemiology Unit and Girona Cancer Registry, Oncology Coordination Plan, Department of Health, Autonomous Government of Catalonia and Descriptive Epidemiology, Genetics and Cancer Prevention Group [Girona Biomedical Research Institute (IdIBGi)], Catalan Institute of Oncology, Girona, Spain.

18: Department of Clinical Sciences, Faculty of Medicine, University of Barcelona,

Barcelona, Spain.

19: University Hospital Marques de Valdecilla – IDIVAL, Santander, Spain.

20: IMIM (Hospital Del Mar Medical Research Institute), Barcelona, Spain.

21: Public Health Division of Gipuzkoa, BioDonostia Research Institute, San Sebastian, Spain.

22: School of Public Health, Athens, Greece.

Supplementary Table 1. List of SNPs included in the genetic risk score

a) GRS24: Genetic Risk Score with 24 SNPs

SNP	Gene	Chromosome	Location	Major allele	Minor allele	Per allele odds ratio	Reference for odds ratio
rs11249433	EMBP1	1	121538815	T	G	1.14	13
rs1045485	CASP8	2	201284866	G*	C	1.04	13
rs13387042	LOC101928278 - LOC105373874	2	217041109	G*	A	1.27	13
rs4973768	SLC4A7	3	27374522	C	T	1.07	13
rs10069690	TERT	5	1279675	C	T	1.08	13
rs10941679	LOC102723839 - RN7SL383P	5	44706396	A	G	1.13	13
rs889312	LOC101928448 - LOC105378979	5	56736057	A	C	1.09	13
rs17530068	LOC105377871	6	81483392	T	C	1.06	13
rs2046210	CCDC170 - ESR1	6	151627231	C	A	1.05	13
rs1562430	CASC21 - CASC8	8	127375606	A*	C	1.11	13
rs1011970	CDKN2B-AS1	9	22062135	G	T	1.06	13
rs865686	LOC105376214	9	108126198	A/T*	G	1.09	13
rs2380205	GDI2 - TRV-TAC3-1	10	5844771	C*	T	1.06	13
rs10995190	ZNF365	10	62518923	G*	A	1.12	13
rs1250003	ZMIZ1	10	79087057	A	C	1.07	13
rs2981582	FGFR2	10	121592803	C	A	1.18	13
rs909116	TNNT3	11	1920716	T*	C	1.05	13
rs614367	LINC01488 - CCND1	11	69513996	C	T	1.13	13
rs10483813	RAD51B	14	68564567	T*	A	1.08	13
rs999737	RAD51B	14	68567965	C*	T	1.08	13
rs3803662	CASC16	16	52552429	C	A	1.21	13
rs6504950	STXBP4	17	54979110	G*	A	1.08	13
rs8170	BABAM1	19	17278895	C*	A	1.06	13
rs2284378	RALY	20	34000289	C*	T	1.04	13

GRS68: Genetic Risk Score with 68 SNPs

SNP	Gene	Chromosome	Location	Major allele	Minor allele	Per allele odds ratio	Reference for odds ratio
rs11552449	AP4B1 - DCLRE1B	1	113905767	C/G	T	1.08	17
rs12048493	OTUD7B	1	149955122	A	C	1.06	18
rs12405132	RNF115	1	145790097	C	T	1.04	18
rs4245739	MDM4	1	204549714	A	C	1.02	17
rs616488	PEX14	1	10506158	T	G	1.05	17
rs6678914	LGR6	1	202218048	G	A	1.01	17
rs72755295	EXO1	1	241870961	A	G	1.15	18
rs12710696	LOC105373455 - MIR4757	2	19121042	C	T	1.03	17
rs1550623	LOC100289479 - CDCA7	2	173348166	T*	G	1.08	17
rs16857609	DIRC3	2	217431785	C	T	1.06	17
rs2016394	DLX2-AS1	2	172108243	C*	A	1.05	17
rs4849887	LINC01101 - LOC105373585	2	120487546	C*	T	1.09	17
rs12493607	TGFBR2	3	30641447	G/T	C	1.07	17
rs6762644	ITPR1	3	4700592	A	G	1.07	17
rs6796502		3	46825376	G	A	1.08	18
rs6828523	ADAM29	4	174925275	C*	A	1.12	17
rs9790517	TET2	4	105163621	C	T	1.03	17
rs10472076	RAB3C - PDE4D	5	58888234	A/T	C	1.05	17
rs13162653	LOC401176	5	16187419	A/G	T	1.03	18
rs1353747	PDE4D	5	59041654	T*	C	1.1	17
rs1432679	EBF1	5	158817075	C	T	1.07	17
rs2012709	SUB1	5	32567626	C	T	1.05	18
rs2736108	TERT	5	1297373	G*	T	1.06	17
rs7707921	ATG10	5	82242227	A	T	1.09	18
rs11242675	FOXQ1 - LINC01394	6	1318643	T*	C	1.05	17

rs12662670	CCDC170	6	151597721	C/T	G	1.22	17
rs204247	RANBP9 - MCUR1	6	13722291	A	G	1.05	17
rs9257408		6	28958443	C	G	1.05	18
rs4593472	LINC-PINT	7	130982362	C	T	1.05	18
rs720475	ARHGEF5	7	144377836	G*	A	1.08	17
rs11780156	MIR1208 - RN7SKP226	8	128182395	C	T	1.1	17
rs13267382	LINC00536	8	116197325	A	G	1.06	18
rs13365225	RPL26P25 - LOC105379377	8	37000965	A	G	1.06	18
rs2943559	HNF4G	8	75505702	A	G	1.13	17
rs6472903	HIGD1AP6 - PKMP4	8	75318066	T*	G	1.12	17
rs9693444	RPL17P33 - LINC00589	8	29652100	C	A	1.05	17
rs10759243	LOC105376206 - LOC105376205	9	107543834	C/T	A	1.06	17
rs11199914	LOC105378523 - RN7SKP167	10	121334387	C*	T	1.04	17
rs11814448	DNAJC1 - ADIPOR1P1	10	22026914	A	C	1.19	17
rs7072776	LOC107984214 - MLLT10	10	21744013	G	A	1.06	17
rs7904519	TCF7L2	10	113014168	A	G	1.06	17
rs11820646	RPS27P20 - LINC01395	11	129591276	C*	T	1.05	17
rs554219		11	69516874			1.27	17
rs75915166		11	69564393	C	A	1.29	17
rs10771399	PTHLH - LOC105369710	12	28002147	A*	G	1.18	18
rs12422552	GNAI2P1 - RPL30P11	12	14260997	G	C	1.05	17
rs1292011	LOC105370003	12	115398717	T*	G	1.08	17
rs17356907	USP44 - PGAM1P5	12	95633983	A*	G	1.09	17
rs11571833	BRCA2	13	32398489	A	T	1.25	17
rs11627032	RIN3	14	92637727	T	C	1.04	18
rs2236007	LOC105370455 - PAX9	14	36663564	G*	A	1.09	17
rs2588809	RAD51B	14	68193711	C	T	1.09	17
rs941764	CCDC88C	14	91374725	A	G	1.06	17
rs11075995	FTO	16	53821379	T	A	1.04	17

rs13329835	CDYL2	16	80616908	A	G	1.09	17
rs17817449	FTO	16	53779455	T*	G	1.06	17
chr17:29230520:D	ATAD5	17	29230520	GGT	G	1.08	18
rs745570	CBX8 - LOC102723961	17	79807926	C	A	1.04	18
rs1436904	CHST9	18	26990703	T*	G	1.04	17
rs527616	PCAT18 - LOC105372035	18	26757460	G*	C	1.05	17
rs6507583	SETBP1	18	44819625	A	G	1.1	18
rs2363956	ANKLE1	19	17283315	T	G	1.02	17
rs3760982	KCNN4	19	43782361	G	A	1.06	17
rs4808801	ELL	19	18460331	A*	G	1.09	17
rs2823093	LOC107985483 - LOC105372739	21	15148511	G*	A	1.06	17
rs132390	EMID - LOC105372985	22	29225488	T	C	1.14	17
rs17879961	CHEK2	22	28725099	C/T	G	1.07	17
rs6001930	MKL1	22	40480230	T	C	1.14	17

Minor allele = risk allele unless major allele is marked with an asterisk

Supplementary Table 2. Linkage disequilibrium between the SNPs indicated in Supplementary Table 1 (Source: https://analysistools.nci.nih.gov/LDlink/?tab=home; accessed: 8 January, 2018)

Chromosome	SNP	rs72755295	rs4245739	rs6678914	rs12048493	rs12405132	rs11249433	rs11552449		
1	**rs616488**	0.0024	0.0003	0	0.0003	0.0011	0.0008	0.0006		
	**rs11552449**	0.0019	0	0.0005	0.003	0.0005	0			
	**rs11249433**	0.0032	0.0002	0.0003	0.0008	0.0012				
	**rs12405132**	0.0001	0.0007	0.0028	0					
	**rs12048493**	0.0022	0.0008	0.0059						
	**rs6678914**	0.0003	0.3417							
	**rs4245739**	0.0006								
**2**	**SNP**	**rs16857609**	**rs13387042**	**rs1045485**	**rs1550623**	**rs2016394**	**rs4849887**			
	**rs12710696**	0.0001	0.0002	0.0002	0.0018	0.0004	0.0041			
	**rs4849887**	0.0004	0.0001	0	0	0.001				
	**rs2016394**	0	0.0005	0.0021	0.0047					
	**rs1550623**	0.0001	0.0044	0.0009						
	**rs1045485**	0	0							
	**rs13387042**	0.0008								
**3**	**SNP**	**rs6796502**	**rs12493607**	**rs4973768**						
	**rs6762644**	0.002	0	0.0012						
	**rs4973768**	0.0011	0.0003							
	**rs12493607**	0.0006								
**4**	**SNP**	**rs6828523**								
	**rs9790517**	0.0016								
**5**	**SNP**	**rs1432679**	**rs7707921**	**rs1353747**	**rs10472076**	**rs889312**	**rs10941679**	**rs2012709**	**rs13162653**	**rs2736108**
	**rs10069690**	0.0001	0.0004	0.0011	0.0004	0.0001	0.0004	0.0001	0.0003	0.0191
	**rs2736108**	0	0	0.0001	0.0031	0.0001	0.0004	0.0021	0	
	**rs13162653**	0.0005	0.0002	0.0006	0.0004	0.0011	0.0001	0.0005		

	**rs2012709**	0	0.0001	0.0002	0.0001	0.001	0.0025	
	**rs10941679**	0.0003	0.0002	0.0014	0.0025	0.0001		
	**rs889312**	0.0002	0.0007	0	0.0001			
	**rs10472076**	0.0001	0.0009	0.0312				
	**rs1353747**	0.0006	0.0014					
	**rs7707921**	0.0003						
**6**	**SNP**	**rs2046210**	**rs12662670**	**rs17530068**	**rs9257408**	**rs204247**		
	**rs11242675**	0.0015	0.0001	0.002	0	0.003		
	**rs204247**	0.0013	0.0018	0.0036	0.0011			
	**rs9257408**	0.0007	0.0007	0.0011				
	**rs17530068**	0.0002	0.0007					
	**rs12662670**	0.1352						
**7**	**SNP**	**rs720475**						
	**rs4593472**	0.0015						
**8**	**SNP**	**rs11780156**	**rs1562430**	**rs13267382**	**rs2943559**	**rs6472903**	**rs13365225**	
	**rs9693444**	0.0001	0.0005	0.0006	0.001	0.0001	0.0002	
	**rs13365225**	0.0005	0.0012	0.0009	0.0003	0		
	**rs6472903**	0.0004	0.003	0.0018	0.0005			
	**rs2943559**	0.006	0.0009	0.0001				
	**rs13267382**	0	0					
	**rs1562430**	0.0005						
**9**	**SNP**	**rs865686**	**rs10759243**					
	**rs1011970**	0	0.0001					
	**rs10759243**	0.0043						
**10**	**SNP**	**rs2981582**	**rs11199914**	**rs7904519**	**rs1250003**	**rs10995190**	**rs11814448**	**rs7072776**
	**rs2380205**	0.0003	0.0002	0.0008	0.0001	0.0007	0.0002	0
	**rs7072776**	0.0024	0.0002	0	0.0001	0	0.0454	
	**rs11814448**	0.0014	0	0.0034	0.0005	0.0013		

	**rs10995190**	0	0.0003	0.0007	0.0001	
	**rs1250003**	0.0027	0.0012	0.0007		
	**rs7904519**	0.0038	0.0007			
	**rs11199914**	0.0006				
**11**	**SNP**	**rs11820646**	**rs75915166**	**rs554219**	**rs614367**	
	**rs909116**	0.0006	0	NA	NA	
	**rs614367**	NA	NA	NA		
	**rs554219**	NA	NA			
	**rs75915166**	0				
**12**	**SNP**	**rs1292011**	**rs17356907**	**rs10771399**		
	**rs12422552**	0	0.0026	0.0001		
	**rs10771399**	0.001	0.0015			
	**rs17356907**	0.0013				
**14**	**SNP**	**rs11627032**	**rs941764**	**rs999737**	**rs10483813**	**rs2588809**
	**rs2236007**	0.0018	0.0005	0.0001	0.0003	0.0009
	**rs2588809**	0	0.0009	0.0044	0.0038	
	**rs10483813**	0.0003	0.0006	0.9821		
	**rs999737**	0.0003	0.0008			
	**rs941764**	0.0001				
**16**	**SNP**	**rs13329835**	**rs11075995**	**rs17817449**		
	**rs3803662**	0.0014	0.002	0.0001		
	**rs17817449**	0.0005	0.0158			
	**rs11075995**	0				
**17**	**SNP**	**rs745570**	**rs6504950**			
	**chr17:29230520:D**	NA	NA			
	**rs6504950**	0.0001				
**18**	**SNP**	**rs6507583**	**rs1436904**			
	**rs527616**	0.0021	0.0001			

	**rs1436904**	0.002		
**19**	**SNP**	**rs3760982**	**rs4808801**	**rs2363956**
	**rs8170**	0.0003	0.0001	0.2609
	**rs2363956**	0	0.0007	
	**rs4808801**	0.0036		
**22**	**SNP**	**rs6001930**	**rs132390**	
	**rs17879961**	0.0005	0.0001	
	**rs132390**	0.0001		

Supplementary Table 3. Categories used in continuous variables (cut points proceed from Maas et al (2016))

**Variable**	**Units**	**Category** [a]									
		**1**	**2**	**3**	**4**	**5**	**6**	**7**	**8**	**9**	**10**
**Age at menarche**	**Years**	≤11	11.0-11.5[b]	11.5-12.0	12.0-13.0	13.0-14.0	14.0-15.0	>15.0			
**Age at first birth**	**Years**	≤19 or no children	19-22	22-23	23-25	25-27	27-30	30-34	34-38	>38	
**Age at menopause**	**Years**	≤40 or premenopausal	40-45	45-47	47-48	48-50	50-51	51-52	52-53	53-55	>55
**Alcohol use**	**Drinks/week**	0.0	0.0-0.4	0.4-0.8	0.8-1.5	1.5-3.2	3.2-5.7	5.7-9.8	>9.8		
**Body mass index**	**kg/m^2**	≤21.5	21.5-23.0	23.0-24.2	24.2-25.3	25.3-26.5	26.5-27.8	27.8-29.3	29.3-31.4	31.4-34.6	>34.6
**Height**	**Meters**	≤1.55	1.55-1.57	1.57-1.60	1.60-1.61	1.61-1.63	1.63-1.65	1.65-1.66	1.66-1.68	1.68-1.71	>1.71

[a] Each category includes right end point but not left end point.

[b] Age at menarche was recorded in accomplished years, so category 2 was empty.

Supplementary Table 4. Sample description according to modifiable and non-modifiable risk factors. Odds ratios (OR) and 95% confidence intervals (CI) are adjusted by age and the remaining factors in the table.

Variable	Category	Breast cancer cases	Controls	OR (95% CI)
**Age (years mean±sd)**		56.4±12.6	59.0±13.2	0.98(0.97-0.99)
**Height (m)**	**≤1.55**	402	447	1(Ref)
	**1.55-1.57**	212	241	0.97(0.75-1.25)
	**1.57-1.60**	379	447	0.89(0.71-1.10)
	**1.60-1.61**	53	50	1.05(0.65-1.70)
	**1.61-1.63**	191	209	0.87(0.66-1.15)
	**1.63-1.65**	193	213	0.86(0.66-1.13)
	**1.65-1.66**	34	34	0.98(0.57-1.69)
	**1.66-1.68**	124	110	1.00(0.72-1.40)
	**1.68-1.71**	78	92	0.77(0.52-1.12)
	**>1.71**	66	61	1.13(0.74-1.75)
**BMI (kg/m^2)**	**≤21.5**	276	319	1(Ref)
	**21.5-23.0**	210	260	0.95(0.72-1.26)
	**23.0-24.2**	198	223	1.16(0.87-1.55)
	**24.2-25.3**	170	195	1.24(0.92-1.68)
	**25.3-26.5**	176	175	1.49(1.10-2.03)
	**26.5-27.8**	164	163	1.55(1.14-2.11)
	**27.8-29.3**	155	150	1.77(1.29-2.43)
	**29.3-31.4**	176	160	1.60(1.17-2.19)
	**31.4-34.6**	119	140	1.36(0.97-1.90)
	**>34.6**	88	110	1.40(0.96-2.04)
**Alcohol consumption (drinks/week)**	**0**	486	540	1(Ref)
	**0.0-0.4**	127	129	0.94(0.69-1.27)
	**0.4-0.8**	132	134	1.03(0.76-1.40)
	**0.8-1.5**	148	171	0.81(0.61-1.08)
	**1.5-3.2**	304	342	0.89(0.71-1.12)
	**3.2-5.7**	265	289	0.83(0.66-1.06)
	**5.7-9.8**	134	166	0.82(0.62-1.10)
	**>9.8**	136	139	1.01(0.74-1.36)
**Family history of breast cancer**	**No first-degree relative affected**	1157	1509	1(Ref)
	**First-degree relative affected**	255	167	2.19(1.76-2.72)
**Age at menarche**	**≤11**	74	100	1(Ref)
	**11.0-11.5**	118	134	1.09(0.71-1.67)
	**11.5-12.0**	333	408	1.05(0.73-1.52)
	**12.0-13.0**	455	463	1.21(0.85-1.74)
	**13.0-14.0**	425	460	1.17(0.81-1.69)

	**14.0-15.0**	246	262	1.19(0.81-1.75)
	**>15.0**	81	65	1.51(0.92-2.50)
**Parity**	**0**	351	355	1(Ref)
	**1**	319	294	1.19(0.78-1.82)
	**2**	674	751	0.97(0.66-1.45)
	**3**	250	293	0.99(0.66-1.49)
	**≥4**	130	206	0.79(0.51-1.21)
**Age at first delivery**	**≤19 or no children**	441	448	1(Ref)
	**19-22**	201	217	1.05(0.70-1.56)
	**22-23**	96	111	0.90(0.57-1.43)
	**23-25**	228	286	0.85(0.57-1.26)
	**25-27**	228	265	0.86(0.58-1.28)
	**27-30**	249	293	0.85(0.57-1.26)
	**30-34**	182	186	0.89(0.58-1.35)
	**34-38**	78	78	0.98(0.59-1.61)
	**>38**	29	26	1.15(0.57-2.32)
**Menopausal status**	**Premenopause**	610	554	1.01(0.76-1.33)
	**Postmenopause**	1122	1356	1(Ref)
**Age at menopause**	**≤40 or premenopausal**	850	893	1(Ref)
	**40-45**	124	198	0.83(0.61-1.13)
	**45-47**	94	84	1.58(1.08-2.30)
	**47-48**	62	107	0.83(0.56-1.22)
	**48-50**	189	243	1.10(0.83-1.47)
	**50-51**	50	34	1.92(1.14-3.23)
	**51-52**	116	126	1.32(0.94-1.85)
	**52-53**	81	61	1.70(1.12-2.58)
	**53-55**	112	113	1.35(0.95-1.92)
	**>55**	54	51	1.63(1.03-2.58)
**Hormone replacement therapy**	**Never**	1574	1692	1(Ref)
	**Ever**	116	135	1.01(0.75-1.36)
	**Not known**	38	68	0.77(0.50-1.19)

Supplementary Table 5. Scores in breast cancer cases and controls, mean±standard deviation

Score	Controls	Breast cancer cases	p
**GRS24**	1.78±0.29	1.85±0.29	<0.001
**GRS68**	3.78±0.36	3.90±0.35	<0.001
**GRS92**	5.56±0.46	5.75±0.45	<0.001
**NMRFS**	0.29±0.45	0.44±0.48	<0.001
**MRFS**	0.27±0.37	0.30±0.36	0.009
**RFS**	0.56±0.62	0.74±0.65	<0.001
**Full model**	6.05±0.76	6.41±0.78	<0.001

GRS24: Genetic Risk Score with 24 SNPs

GRS68: Genetic Risk Score with 68 SNPs

GRS92: Genetic Risk Score with 92 SNPs

NMRFS: Non-modifiable Risk Factor Score

MRFS: Modifiable Risk Factor Score

RFS: Risk Factor Score

Supplementary Table 6. Pearson correlation coefficient among genetic scores, modifiable risk factor score, nonmodifiable risk factor score and full model

	GRS68	GRS24	GRS92	NMRFS	MRFS	RF
**GRS24**	0.01					
**GRS92**	0.78	0.64				
**NMRFS**	0.05	0.04	0.07			
**MRFS**	<-0.01	0.01	<0.01	0.22		
**RF**	0.04	0.03	0.05	0.82	0.74	
**Full model**	0.49	0.40	0.63	0.58	0.58	0.81

GRS24: Genetic Risk Score with 24 SNPs
GRS68: Genetic Risk Score with 68 SNPs
GRS92: Genetic Risk Score with 92 SNPs
NMRFS: Non-modifiable Risk Factor Score
MRFS: Modifiable Risk Factor Score
RFS: Risk Factor Score

Supplementary Table 7. Sensitivity analysis on imputation results: Relationship between breast cancer and GRS24 (in deciles) according on whether GRS24 was obtained by genotyping or by imputation: Odds ratios (OR) and 95% confidence intervals

GRS24 decile	OR in the whole sample	OR in 1138 cases and 1239 controls with available genotype	OR in 594 cases and 671 controls without available genotype
**1**	1 (reference)	1 (reference)	1 (reference)
**2**	1.20 (0.89 – 1.63)	0.95 (0.66 – 1.37)	1.43 (0.83 – 2.47)
**3**	1.13 (0.83 – 1.53)	1.04 (0.72 – 1.49)	0.85 (0.49 – 1.49)
**4**	1.54 (1.14 – 2.08)	1.27 (0.88 – 1.83)	1.28 (0.74 – 2.21)
**5**	1.31 (0.96 – 1.77)	1.38 (0.96 – 1.99)	2.00 (1.16 – 3.43)
**6**	1.58 (1.17 – 2.14)	1.23 (0.85 – 1.77)	1.89 (1.10 – 3.24)
**7**	1.49 (1.10 – 2.01)	1.21 (0.84 – 1.74)	1.66 (0.97 – 2.86)
**8**	1.93 (1.42 – 2.61)	1.78 (1.24 – 2.57)	1.46 (0.85 – 2.51)
**9**	1.83 (1.35 – 2.48)	1.58 (1.10 – 2.28)	2.40 (1.39 – 4.14)
**10**	2.34 (1.73 – 3.17)	1.94 (1.35 – 2.80)	2.79 (1.62 – 4.83)

Supplementary fig. 1. ROC curves of genetic and risk factor scores – breast cancer relationship.

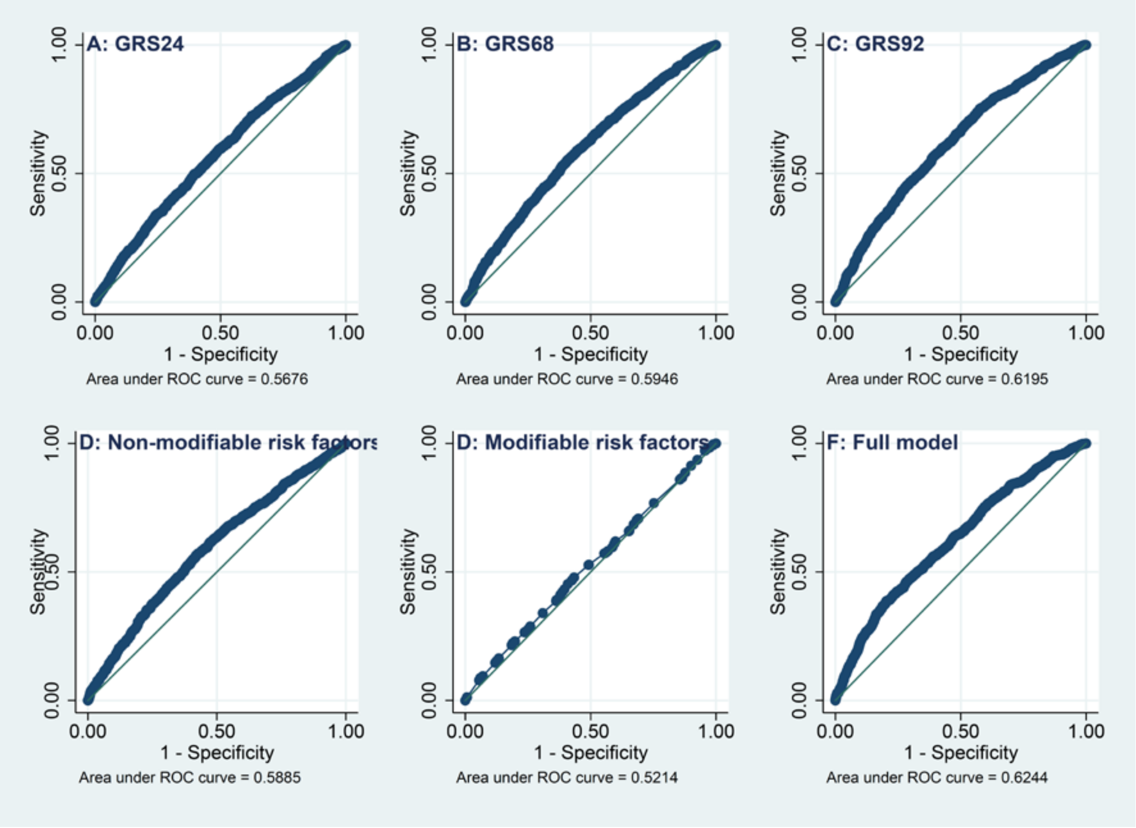

Supplement: Supplementary file 1 — Supplementary material [file 41598_2018_20832_MOESM1_ESM.pdf]
